# Special Issue of the 4th Symposium of the Canadian Society for Virology 2022

**DOI:** 10.3390/v15071512

**Published:** 2023-07-06

**Authors:** Vanessa Meier-Stephenson, David Evans, Tom Hobman, David Marchant, Ryan Noyce, Maya Shmulevitz

**Affiliations:** 1 Department of Medicine, Division of Infectious Diseases, University of Alberta, Edmonton, AB T6G 2G3, Canada; 2 Department of Medical Microbiology and Immunology, University of Alberta, Edmonton, AB T6G 2R3, Canada; devans@ualberta.ca (D.E.); thobman@ualberta.ca (T.H.); marchant@ualberta.ca (D.M.); noyce@ualberta.ca (R.N.); shmulevi@ualberta.ca (M.S.); 3 Li Ka Shing Institute of Virology, University of Alberta, Edmonton, AB T6G 2R3, Canada; 4 Department of Cell Biology, University of Alberta, Edmonton, AB T6G 2H7, Canada

In this Special Issue of *Viruses*, we showcase some of the fascinating and diverse virology being undertaken in Canada that was presented at the 4th Symposium of the Canadian Society for Virology 2022 [1,2,3,4,5]. This symposium was hosted by the University of Alberta and the Li Ka Shing Institute of Virology in Edmonton, Alberta, Canada, and had a record number of attendees, all eager to talk virology and share new and evolving ideas. During our publication window for submissions with *Viruses*, we accrued five manuscripts to highlight research that focuses on human, animal, and bacterial viruses.

Beginning with work by Hare et al. [1], this team builds on prior research examining the innate immune response to viral fusion with the cell membrane. They use a reovirus fusion protein, p14 in lipoplexes introduced exogenously (representing viral fusion) and expressed endogenously (representing cell–cell fusion/syncytia formation) to explore the differential antiviral signalling pathways that become stimulated following membrane perturbations.

Yousefi et al. [2] investigate the interplay between the APOBEC enzymes in relation to their ability to restrict HIV replication. More specifically, they explore the link between APOBEC3H (haplotype I), highlighting the fact that while it has the unique ability to resist HIV Vif protein-induced degradation, it can succumb to more rapid degradation when complexed with APOBEC3F. Interestingly, APOBEC3F expression is increased with APOBEC3H, and only in the presence of APOBEC3H does the APOBEC3F gain the added ability to restrict HIV.

Next, turning towards Mpox (monkeypox at the time of submission; MPXV), we highlight the important proof-of-principle work by Noyce et al. [3]. Here, researchers vaccinated cynomolgus macaques with a novel recombinant chimeric horsepox virus (rcHPXV; known as TNX-801) and show that their vaccine produces protective immunity following a lethal challenge with Mpox virus, Zaire strain. The vaccine was protective against severe disease outcomes as well as the development of skin lesions. This work provides the first steps in showing both the safety and efficacy of this viral platform and that its timely development may give a head start for the next poxvirus-related outbreak.

Along similar lines in vaccine research, Aubrey et al. [4] highlight some important research conducted in the One Health realm in which vaccination options are investigated for H1N2 and H3N2 influenza strains in swine. Their work demonstrates the application of their previous elastase-dependent bivalent live-attenuated vaccine derived from two Canadian swine influenza A isolates (from 2015 and 2016) and shows that their efficacy extends to two more current isolates of drifted non-homologous H1N2 and H3N2 strains. This again fills an important niche in the protection of these animals from swine flu and potential spillover events that may occur as part of outbreak scenarios.

Finally, Gomez et al. [5] describe the application of bacteriophages in our food industry but with practical considerations to ensure their optimized and rigorous use. They focus on the bacteriophage P100, used against *Listeria monocytogenes*, a bacterial pathogen that has been linked with prior contaminations of processed sandwich meats. Their team reports on the importance of bacteriophage stability under various stressors, including desiccation, elevated temperature, and low pH, mimicking various food preparation environments, and uses these conditions to both quantify those effects and select for stress-resistant bacteriophages. Their work may have broader applications to other food industry phages as this field continues to evolve.

Overall, the collection of these manuscripts is, of course, only a small sampling of the great work being carried out by Canadian virologists and highlights a diverse range of studies, from fundamental understanding to practical applications to therapeutics development.
